# An evaluation of the benefits to a UK Health Care Trust working in a partnership with a hospital in Northern Uganda: International partnership working in mental health

**DOI:** 10.1186/s12992-015-0134-8

**Published:** 2015-12-22

**Authors:** Ben Hague, Jenny Sills, Andrew R. Thompson

**Affiliations:** Clinical Psychology Unit, University of Sheffield, Western Bank, Sheffield, S10 2TN South Yorkshire UK

**Keywords:** International health partnerships, Mental health, Qualitative methods, Thematic analysis, Africa

## Abstract

**Background:**

Despite the worthy intentions of international health partnerships between high-income countries and countries with developing economies, the tangible benefits are rarely evaluated, limiting the assessment of the achievements of such collaborations.

**Methods:**

The present study used longitudinal qualitative methods to examine the individual and organisational benefits of a partnership between a National Health Service (NHS) mental health Trust in the United Kingdom and a mental health referral hospital in Northern Uganda. Benefits to UK staff and organisational development were benchmarked against an existing framework of healthcare competencies.

**Results:**

Partnership involvement was beneficial to UK staff, by increasing awareness of diversity, and in enhancing ability to work flexibly and as a team. There were clear benefits expressed with regards to the partnership having the potential to enhance organisational reputation and staff morale.

**Conclusions:**

The findings from this study demonstrate that international partnerships are experienced as being of tangible value for healthcare staff from high-income countries, providing opportunities for the development of recognised healthcare competencies. In this study there was also some evidence that staff involvement might also provide wider organisational benefits.

## Background

Africa holds 11 % of the world’s population, has 24 % of the world’s global disease, and yet has only 3 % of the world’s health workers, with few of these working in mental health [1]. Analyses of global physical health patterns have indicated the role played by mental health, not only as a co-morbid condition existing alongside physical disease, but also as a significant risk factor for the development of physical illnesses [2]. Consequently, supporting health care in Africa has been placed at the heart of the United Nations Economic and Social Council’s global public health agenda [3]. The World Health Organisation has also placed great emphasis on the development of global health strategies aimed at redressing the scarcity of mental health provision and funding in low-income and middle-income countries (LMICs) [4, 5]. One of the many challenges to overcome in developing effective mental health treatments in LMICs are the cultural and staffing barriers appropriate to the host region [6, 7], and therefore partnership working and the balanced exchange of knowledge between high-income and LMICs have become a priority in global health strategies [3, 6].

According to the UK-based Tropical Health and Education Trust (THET), a major funder of international health care partnerships, the aims of international health partnerships are two-fold: Firstly to support healthcare development in LMICs, and secondly to provide benefits to the healthcare sector within the partner country [8]. Yet despite numerous partnerships established between countries with developed healthcare systems and Africa, there have been few formal evaluations of the benefits provided to stakeholders. A database search (using PubMed and Scopus) found two studies that systematically reviewed international partnership benefits; concluding that (a) a lack of standardised methods exist in reporting benefits to both partners [9], and (b) the effects on the partner from the developed health care system are frequently undermined by poor methodological rigor, or simply go unmonitored [10]. This is regrettable as mapping the experiences of staff to development or organisational competencies has the potential to provide support for the continuation of partnership working, whilst facilitating the transfer of benefits within the wider developed countries healthcare system [11–13]. Moreover, many established healthcare providers are currently operating within stringent economic conditions, and it is therefore essential that international partnerships continue to monitor and evaluate the impact and benefits upon both partners [14]. Clearly there is a need to measure wider individual and organisational benefits of partnerships, assessing the value of international health partnerships for commissioners, managers, and staff across collaborating countries, in order to sustainably developing global policy in human resources [1]. White and Sashidharan [6] commented upon global mental health strategy in their recent editorial in The British Journal of Psychiatry, stating that: “Only by engaging in critical reflection about how mental health services are designed and delivered in both high-income countries and LMICs can we foster a global mental health that is truly global” (p.416).

One method of assessing the impact of international partnerships upon staff and organisational development is to benchmark developing competencies against existing frameworks of healthcare competencies. Within the UK, the National Health Service (NHS) has used the ‘Knowledge and Skills Framework’ (KSF) to measure staff competencies and skills across health care practitioners [15]. The KSF has been used as a method of appraising continued professional development (CPD). Longstaff [16] used the KSF framework to evaluate international partnerships, demonstrating that the knowledge and skills gained from international partnership working fitted well with the core dimensions of the KSF, particularly in relation to the domains of c*ommunication*, *personal development*, and e*quality and diversity*. Consequently, the development of a toolkit was commissioned to provide NHS employers with a framework for collecting evidence of the potential organisational benefits of staff involvement in international partnership working [17]. However, to the authors knowledge there have been no studies published that have used this framework and it remains unclear as to whether the potential organisational benefits described in previous reports [16] have been replicated.

### The Sheffield-Gulu partnership

The Sheffield-Gulu partnership is a mental health partnership that encompasses UK National Health Service (Sheffield Health and Social Care NHS Foundation Trust: SHSC) and non-governmental organisations from the voluntary, faith and charity sectors in both the UK and in Uganda [18]. Formally established in 2012, the primary intention of the link was to form working relationships between SHSC, Gulu Regional Referral Hospital, and mental health service user groups in both Uganda and the UK, in order to improve mental health service provision in both countries. Initial visits to Gulu by SHSC health professionals served to establish formal working relationships with interested stakeholders, forming partnerships with the Mental Health Unit at Gulu Regional Referral Hospital in 2012, and Mental Health Uganda (a non-profit community mental health organisation) in 2013. The agreed role of Gulu Regional Referral Hospital was to support a needs assessment at the Mental Health Unit, which resulted in staff leads being identified to visit the UK to observe practices, shadow staff, and receive specialist NHS training designed to enable staff to improve communication, safety and care provided to mental health service users in Gulu. The role of Mental Health Uganda was to collaborate with SHSC on the development of a Mental Health Awareness programme and to support continued funding applications for the Sheffield-Gulu link.

Further funding was secured from a Commonwealth Fellowship scheme and private charitable funding that enabled the partnership to continue. The partnership used this funding in order to deliver further reciprocal visits between partnership stakeholders, and mental health awareness training for Hospital staff, with a focus on improving patient safety at Gulu Referral Hospital.

### The present evaluation

Evaluating the benefits to the healthcare systems in both collaborating countries within the partnership is essential. However, the present evaluation deliberately focuses on UK benefits of the Sheffield-Gulu partnership because of the need to demonstrate the benefits in a climate where UK health providers are under pressure to demonstrate the value of any additional activity; and as previously stated there has been less attention paid to the benefits for partners from developed healthcare systems. The present study sought to address the following aspects of the partnership:

1. To identify and describe how the partnership has contributed to personal and professional development for NHS staff from the UK side of the partnership.
2. To identify and describe how the partnership has contributed to organisational benefits for the NHS.

## Method

### Design

A longitudinal qualitative design was employed to systematically collect data from SHSC partners of the Sheffield – Gulu Partnership, using thematic analysis. Thematic analysis was chosen over other qualitative methods due to its ability to capture in-depth information about a specific context with a relatively small sample [19, 20]. Data collection was divided into two phases in order to describe both the individual and organisational benefits to the NHS: First, individual benefits were assessed through individual interviews before and immediately after a visit to Gulu. Second, organisational benefits were evaluated through focus groups with members of the Sheffield partnership team following an initial visit to Gulu and one-year post-visit. A one-year timeframe was chosen as it maps onto the typical period considered during NHS staff reviews, as well as enabling staff to have had sufficient reflection on the transferability of learning post-visits. Clinical governance approval was obtained from SHSC and all participants provided written consent.

### Participants

All members of SHSC staff involved in the Gulu-Sheffield partnership agreed to participate (*N =* 8) in the study and took part between June 2012 and June 2013. The most recent members to join the partnership (*n* = 2, one male, one female, both aged 45) were interviewed before travelling to Uganda and on their return. In addition, the existing members of the UK staff team (*n =* 6, five females, one male, mean age = 43.8, range in years = 29) participated in two focus groups set apart by twelve months. The participants had varying professional backgrounds (social work, nursing, and occupational therapy), and ranged in number of years experience post-qualification (8–27 years), and level of seniority (partnership lead, ward staff, and a senior Trust executive). Participants also ranged in their number of prior visits to Gulu (between one and four visits).

### Data collection

Semi-structured interview schedules were used in both individual and focus group interviews. The schedules were developed by all authors, and informed by the KSF competencies described in previous research [16, 17].

Individual interviews were conducted with two participants one week before their first visit to Gulu (entry interviews), and within one month of returning (exit interviews). Entry interviews were semi-structured to explore background, preparation, aims and personal and professional goals for the visit. Exit interviews were semi-structured to review the achievement of goals identified during the entry interviews, with the encouragement of wider reflection on the outcome of the visit. Each individual interview lasted approximately 30 min, and was co-led by the first and second authors. The interviews took place at the participants’ place of work or the University of Sheffield. Interviews were recorded using digital equipment. An independent research assistant transcribed all four interviews.

Focus groups were conducted with the established SHSC partnership team immediately after and one year following a 3-week visit to Gulu. The initial focus group explored their preparation, aims, and experiences of a 3-week trip to Gulu. The follow-up focus group was structured to identify how membership in the partnership was of benefit to the organisation (NHS) one year on. Open discussion was also facilitated within the focus groups. Each focus group lasted between 60–90 min, and was led by the third author, and the first and second authors made notes and a recording of the interview.

### Data analysis

Thematic analysis was used to analyse the data, as it provides a method for identifying, analysing, and reporting descriptive patterns within texts [19]. Initial codes were generated from the four transcripts from interviews at both time points. Initial codes were defined as themes when there was data derived from at least two participants. Superordinate themes were identified in accordance with a priori dimensions drawn from the Knowledge and Skills Framework [15]. There are several variations of the KSF dimensions and Table 1 shows those defined by the developed toolkit [17] that were used as a thematic template in this study. ‘A priori themes’ of the KSF dimensions were applied to both focus groups and the exit interviews only in phase one.

**Table 1:** KSF dimensions according to NHS staff competencies toolkit [15]

KSF dimension	Description
Communication	Overcoming barriers to communication/communicating difficult messages
Personal and people development	Personal confidence, and professionally your experience of mentoring others or teaching and training
Equality and diversity	Cultural awareness and your ability to relate to people from diverse backgrounds
Capacity and capability	Working with others to evaluate services/ your own work through audit etc.
Services & project management	Confidence in your ability to work with others on collaborative projects
Developing leadership skills	Problem solving skills, championing innovation and change, awareness of wider management issues at work.

An independent research assistant provided inter-rater validation of the analysis to ensure that the method of arriving at the final themes had been transparent and that the process had been sufficiently thorough [20]. Validation involved showing how the data from all of the transcripts was linked to the final themes and ensuring that significant portions of the data were not left unaccounted for. Discrepancies identified as a result of the inter-rater process were resolved by merging codes that were similar and likewise by refining codes that were consistently different. The process of validation recurred until an agreement rate between the first author and the inter-rater exceeded 80 % [21]. In the present evaluation, the agreement rate exceeded 80 % on the third attempt for entry interviews and the second attempt for the exit interviews. Participant validation was sought by circulating the results to all participants with a request for feedback on the themes. No feedback was received and it was assumed that participants were content with the accuracy of the findings.

## Results

### Entry interviews

Before visits to Uganda, three themes emerged regarding perceived power imbalances between the UK and Ugandan partners. Moreover, participants discussed managing negative experiences and the value of preparation by SHSC.

#### Colonialism and power

Past colonialism and power affected how participants perceived their engagement with Gulu partners:

*“I’d rather build up a bit of a relationship with them first. There are lots of white people that go in to their country and dictate a lot of changes” Participant A*

Consequently, the importance of equal choice in the face of a power imbalance was reported:

*“It’s a case of me going in and showing my skills… if they want to embrace them that’s their choice entirely” Participant B*

#### *Preparation*

Participants experienced the preparation for the trip as valuable and positive, but feelings of ignorance towards the history of Gulu were raised:

*“I feel I’ve learnt a lot before I’ve even gone there. I’d class myself as quite culturally aware before (the trip) but learning about Uganda highlights that you aren’t” Participant A*

#### *Managing experiences*

Participants’ anticipated being shocked by what they would see in Gulu:

*“From what I heard you see some quite traumatic things taking place. You’re not able to do anything?” Participant B*

Consequently, both the reflective logs and debriefs were recognised as arenas to support them to cope with any shocks:

*“People have seen things they found quite traumatic. If we could have a good talk about it at the end of the day… those sorts of things will go in the journal as well” Participant A*

### Exit interviews

Following the trip to Gulu, participants raised emerging respect for flexible ways of working and the confidence to adapt practices in the UK after witnessing economical use of resources in Uganda. The importance of building relationships was developed through the trip and the value of preparation was revisited to manage cultural shocks on the visit. Three salient a priori themes emerged according to KSF competencies:

#### Personal and people development

Participants reported that international work had made a positive impact on their UK roles, and they felt greater motivation for two-way learning between staff and service users:

*“I am not as paternalistic. I now ask what are you going to do about it? Participant B*

Reporting on the use of debriefs and reflective logs, participants felt they had supportive and limiting functions:

*“Because of some of the traumatic stuff we saw that debrief at the end of the day was essential. It was quite relaxed” Participant A*

*“I had to make the decision to be truly honest, or if people will see the log. I decided to calm it down, but be honest” Participant B*

#### Equality and diversity

After the visits, participants demonstrated a respect for the diversity they encountered, challenging preconceptions they held before the trip:

*“Before I went I couldn’t believe they give people this medication and do it this way, but when I was there I could understand why they have to do it that way because of limited resources” Participant B*

Moreover, participants commented on how the preparation had improved their experiences:

*“Because the preparation was perfect when I arrived there I didn’t feel shocked. There were a couple of things they couldn’t really prepare you for but yeah I think the preparation was brilliant” Participant A*

Participants spoke about how the experience of different cultures has helped adapt practices on the trip and back in the UK in training roles:

*“I talked about shaking hands there, and how it can feel uncomfortable. This helped the class think about how we use space and touch on the wards, and how important it is to ask (pause) touching people generally in our culture is okay” Participant A*

#### *Communication*

Participants spoke about their ability to build relationships with Gulu partners in a relatively short visit:

*“In just two weeks I’ve managed to build up a good enough relationship with some of the staff to be brutally honest to them.” Participant B*

However, suspicion remained in the participants’ mind post-visit with regards to practices in Gulu that could not be communicated:

*“I have my own thoughts on what may have happened there but no one told us any different. I feel suspicious, but I’ll probably never find out” Participant A*

Relating to the KSF, a priori themes around *developing leadership skills* or *project management* or *capacity and capability* were not apparent immediately after the trip.

### First focus group

Immediately following a trip to Gulu, participants raised feelings of anxiety regarding several aspects of the partnership including inequality between UK and Ugandan partners, barriers to partnership development, and ethical dilemmas regarding power and safety in Ugandan hospital wards. These themes fit with three of the KSF competencies and are described below.

#### Services and project management

Despite building positive relationships, all participants reported a strong sense of inequality in the partnership:

*“Not starting from an equal with inequality around access to funds. There is an inherent power in that” Participant D*

*“It is not a two-way street, do we need to acknowledge the inherent power imbalance and adhere to a guiding set of principles?” Participant F*

#### Capacity and capability

Also, participants described fear of unsustainability through a lack of resources and funding:

*“I suppose there is a fear that the work we have done will not be supported by the hierarchy in Gulu. It is frustrating that the processes are there but they are not getting the support to implement them” Participant D*

*“Makes you feel quite helpless when it is dictated by resources (pause) you just hope it is sustainable” Participant C*

Consequently, participants sensed that more needed to be done at a strategic level:

“*Unsure what the partnership will look like (pause) a feeling that it will take time, not sure who the main players (in Gulu) are at a strategic level?” Participant H*

*“Learning still needs to happen about the hospital infrastructure. It is very hierarchical and we are unsure who the primary link members are” Participant F*

#### *Communication*

A lack of communication was noted between UK and Gulu stakeholders that impacted on the relationships:

*“There a was a general lack of awareness about our trip (pause) we need to give staff more information as to why we were there” Participant E*

*“Relationships seem fragile at the minute (pause) what did the staff make of us going into the hospital? I got a sense of bemusion and being guarded” Participant C*

Moreover, an ethical dilemma was acknowledged between challenging unsafe practice versus the inherent power imbalance recognised from the trip:

*“It is challenging to be a nurse and seeing differences in ways of working that do not sit right. To be an observer can be hard” Participant H*

*“Need to share concerns with staff as there is a tension because some practices I did not agree with. So it is how you do this?” Participant E*

### Follow-up focus group

From the interviews, four main themes became evident one year on. Participants described key developments in two-way learning between partner countries, an evolving identity for the partnership within the UK Trust, confidence in their ambassadorial roles and a true sense of partnership through shared inspiration. These themes fit with four of the six KSF competencies: *services and project management*, *capacity and capability*, *leadership skills* and *personal and people development* [17].

#### Services and project management

A true sense of partnership had emerged in the Sheffield Gulu partnership, with previously reported themes regarding inequality disappeared one year on:

*“Initially it was one-way. Undoubtedly now it is an actual partnership” Participant D*

*“Up to June 2012 it did not feel like a partnership. Now it feels constant” Participant G*

In particular, all participants were inspired by the input provided by Mental Health Uganda; a dedicated group of people who had been patients at Gulu Mental Health Unit. Developing healthcare that is informed by users of the service is known as ‘service user involvement’ in the UK, with study participants unanimously feelings that this level of involvement was something for the NHS to learn from:

*“Witnessing service user involvement at a different level, it blows your mind” Participant D*

*“The UK model is too paternalistic the governance can minimise inspiration and creativity that we saw in Gulu” Participant G*

#### Capacity and capability

A fear of unsustainability in SHSC partners immediately post-visit had changed to a growing sense of sustainability through clarity of roles, and secured funding:

*“Since securing more funding the executive lead has been very important for driving the project forward. Clear roles and increased clarity” Participant E*

*“The partnership has legitimacy now (pause) a place” Participant G*

#### *Leadership skills*

From continued partnership involvement, participants reported being more competent in communicating difficult messages, addressing differences in practice on the wards, and some participants had adopted an ambassadorial role within their UK roles:

*“We are able to challenge each other when we do not agree (pause) better at diplomacy and checking out and challenging colleagues” Participant F*

*“Built confidence in public speaking as we had forums where we presented to the trust. We made a conscious decision to widen out to the broader trust” Participant D*

At a management level, participants reported being more able to consider resource management in the NHS, and developed skills in problem solving in their roles:

*“Encouraging people to be more resourceful (laughs) and less whingeful” Participant E*

*“Lateral thinking that can now be applied to our senior management meetings from experiences in Gulu” Participant D*

#### Personal and people development

Participants were more confident in continuing to provide mentoring when back in UK roles:

*“Much more interested in mentoring style with SHSC staff. The use of encouragement in supervision and coming from a let’s work this out together” Participant E*

*“Still have on going contact with partners from Uganda on going emails and mentoring roles on a day-to-day basis with staff” Participant F*

In general, all participants felt that the partnership had developed them personally, gaining a strong sense of living their values:

*“The partnership has made me a better person” Participant F*

*“It gets under your skin affects everything that you do. It has changed me and inevitably changed my professional work, living your values in practice nurturing staff you know the core human values” Participant D*

## Discussion

The present evaluation aimed to assess the benefits to individuals and organisations involved in international health partnerships between the UK and Africa, building on a previously developed toolkit [17]. In this study, participants from the UK side of a Sheffield-Gulu partnership described competency development in several areas of the KSF, and this can be seen in an evolving process of continued professional development (CPD). Collated from reports in both phases immediately following a trip to Gulu, participants described developing KSF competencies in *communication*, *equality and diversity* and *capacity and capability,* supporting the findings of a previous international partnership evaluation [16]. Participants’ reported having gained a sense of respect of different cultures and a greater confidence to adapt practice and consider resource management domestically. In the context of current financial austerity, in which services are asked to be more economical with resources, partners’ ability to greater consider and implement resource management in the domestic roles has implications for the cost-saving potential of engaging staff in international health projects.

The longitudinal design used in the present study illustrated an evolving process of skills development of staff engagement in international health partnerships. After continued involvement in the Sheffield-Gulu partnership, participants described competency development in *people development, leadership skills*, and *project management*. A wish for partnership working amidst fragile relationships and a fear of unsustainability were replaced by an identity and legitimacy in the partnership one year following participation. Despite the inherent power imbalance, which was acknowledged by participants, continued involvement in the Sheffield-Gulu partnership was seen as having learning, most notably in terms of inspiration taken from the service user involvement in Uganda to implement in the UK. This was an example of two-way learning and reflected the learning in action during the repeated visits (and preparation for visits) to Gulu, which resulted in a true sense of working in partnership. Moreover, participants described a developed sense of personal and professional integrity and commitment to adhering to the partnership model in their NHS roles. An ethos of working creatively and in partnership with colleagues and service users had clearly spread to other areas of people’s work. Specific skills developments were demonstrated in funding applications, dissemination to colleagues and mentoring roles both within the partnership and in their domestic roles.

### Implications and recommendations

From the findings of the evaluation, important implications for international health partnerships can be made. The present study showed the value of formal preparation and goal setting to acknowledge anxieties around cultural ignorance and power inequity apparent in international health partnerships. A formalised process of entry interviews might be established so as to ensure that individuals entering an international partnership have clear goals, which might be appraised within the personal appraisal process. Equally, tools such as peer reviews on trips and personal reflective logs might enhance the capture of learning/developmental issues experienced on visits.

For more established partners, on-going contact with collaborating partners and mentoring roles after initial visits should be encouraged to develop competencies in *people development* and *leadership*. Demonstrating competency development in important areas such as project management as described in the present study has important implications for securing continued funding of international health partnerships, which are under particular scrutiny in the current climate of economic austerity. International partnerships organisations need to better evaluate their work and to consider how evaluation and research methods can be built into routine partnership practice.

### Strengths, limitations, and future directions

This evaluation is the first study to explore the effectiveness of a staff appraisal toolkit [17] in gathering relevant data about knowledge and skills gained by healthcare professionals engaged in international projects and how this may benefit their wider organisations. It demonstrates how rich and useful information can be gained on partnership working from relatively small samples. A second strength of the study is the use of a longitudinal design, which enabled time for the participants to reflect on the personal and organisational learning and changes that had resulted from engagement in the health care partnership over a 12-month period. This suggested that a variety of KSF competencies developed at different rates over time.

There are some important limitations of the present study that (a) should be acknowledged, and (b) may serve to suggest directions for future research: First, evaluating one side of the partnership is an acknowledged limitation of the present study. As stated in the introduction, the decision to focus on UK partners was based to the stated economic climate of austerity and pressure for UK health providers to justify funding in this context. However, evaluation of the benefits to the Ugandan partners of the Sheffield-Gulu Partnership is ongoing and integrating these findings is an essential next step. Second, the study is by its very nature based on a small and specific sample that may limit the transferability of the findings to other partnerships. As participants did not provide any feedback on the findings it is unclear as to whether participant validation was actually achieved. Finally, whilst we attempted to guard against participants providing an overly positive account of the partnership by involving independent interviewers this cannot be ruled out and replication is required with further studies with other partnerships. Future research in this area might also deploy longer follow-up periods and use observational methods to examine objectively change in staff competencies.

## Conclusion

This evaluation has added to existing evaluations of international partnerships using formal staff development tools [16, 17] and provides some initial evidence of the further benefits of international health partnership work in mental health. The study has been innovative in using interviews to gain access to healthcare workers experience of involvement in partnership work before and immediately after their first involvement. This approach has demonstrated that such partnerships provide fertile ground for the development of continued professional development. The findings from this study provide further evidence that international partnerships can have tangible value for high-income countries as well as for developing countries, providing skills transfer to healthcare staff that have the potential to provide organisational benefit.

## Abbreviations

LMIC: Low-income and middle-income country
KSF: Knowledge and skills framework
NHS: National Health Service
SHSC: Sheffield Health and Social Care NHS Foundation Trust
THET: Tropical Health and Education Trust
